# Partial Conservation between Mice and Humans in Olfactory Bulb Interneuron Transcription Factor Codes

**DOI:** 10.3389/fnins.2016.00337

**Published:** 2016-07-20

**Authors:** Nana Fujiwara, John W. Cave

**Affiliations:** ^1^Burke Medical Research InstituteWhite Plains, NY, USA; ^2^The Feil Family Brain and Mind Research Institute, Weill Cornell MedicineNew York, NY, USA

**Keywords:** differentiation, transcription factor, Tyrosine hydroxylase, Calretinin, Calbindin, olfactory bulb

## Abstract

The mammalian main olfactory bulb (OB) has a large population of GABAergic inhibitory interneurons that contains several subtypes defined by the co-expression other neurotransmitters and calcium binding proteins. The three most commonly studied OB interneuron subtypes co-express either Calretinin, Calbindin, or Tyrosine hydroxylase (Th). Combinations of transcription factors used to specify the phenotype of progenitors are referred to as transcription factor codes, and the current understanding of transcription factor codes that specify OB inhibitory neuron phenotypes are largely based on studies in mice. The conservation of these transcription factor codes in the human OB, however, has not been investigated. The aim of this study was to establish whether transcription factor codes in OB interneurons are conserved between mice and humans. This study compared the co-expression of Foxp2, Meis2, Pax6, and Sp8 transcription factors with Calretinin, Calbindin, or Th in human and mouse OB interneurons. This analysis found strong conservation of Calretinin co-expression with Sp8 and Meis2 as well as Th co-expression with Pax6 and Meis2. This analysis also showed that selective Foxp2 co-expression with Calbindin was conserved between mice and humans, which suggests Foxp2 is a novel determinant of the OB Calbindin interneuron phenotype. Together, the findings in this study provide insight into the conservation of transcription codes for OB interneuron phenotypes between humans and mice, as well as reveal some important differences between the species. This advance in our understanding of transcription factor codes in OB interneurons provides an important complement to the codes that have been established for other regions within the mammalian central nervous system, such as the cortex and spinal cord.

## Introduction

Neural circuit development requires the assembly of many neurons with distinct phenotypes. Molecular mechanisms that direct neuronal progenitor differentiation are critical for providing the phenotypic diversity necessary for circuit development. Neuronal differentiation is marked by the expression of transcription factors that divide neural progenitor domains into distinct territories. The expression of additional transcription factors within these domains further refines and subdivides these territories into regions that produce progenitors for a subset of phenotypes. The combination of transcription factors used to specify progenitors for a given phenotype is referred to as a transcription factor code.

The mammalian main olfactory bulb (OB) has a large and diverse population of inhibitory interneurons. The OB is the initial processing site of odorant sensory information within the central nervous system. In the OB glomerular layer, odorant information is relayed from olfactory sensory neuron axons to mitral/tufted cells. The relay is modulated by glomerular layer inhibitory neurons. The transmission of odorant information from mitral/tufted cells to other cortical regions is regulated by inhibitory neurons in the granule cell layer. Nearly all OB inhibitory neurons are GABAergic, but there are many sub-types that are defined by the differential co-expression of other neurotransmitters, neuroactive peptides, and calcium binding proteins (Kosaka et al., 1998; Panzanelli et al., 2007; Parrish-Aungst et al., 2007). The three most commonly studied interneuron phenotypes are defined by the expression of either Calretinin or Calbindin calcium binding proteins, or Tyrosine hydroxylase (Th; the rate-limiting enzyme for dopamine biosynthesis).

**Figure 1:**
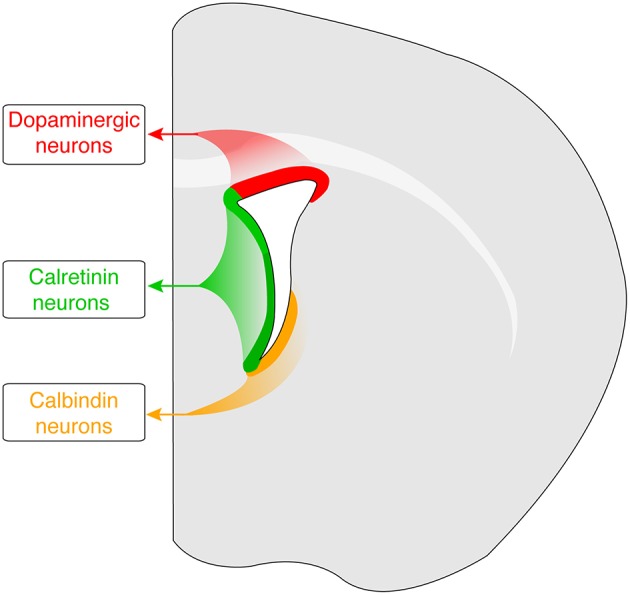
**Spatial origins of progenitors for Th-, Calbindin-, and Calretinin-expressing OB interneurons in the mouse adult SVZ**. Dopaminergic (Th-expressing) progenitors are generated from the dorso-lateral region (shown in red). Progenitors of Calretinin-expressing OB interneruons are produced in the medial and dorsal regions (shown in green), whereas Calbindin-expressing progenitors are generated in the dorsal and lateral regions (shown in orange).

Our current understanding of transcription factor codes that specify OB inhibitory neuron phenotypes is largely based on studies in mice. OB interneuron progenitors in mice are generated by neural stem cells in the subventricular zone (SVZ) of the lateral ventricle [reviewed in Cave and Baker (2015). The post-natal and adult subventricular zone is partitioned into several domains defined by the expression of distinct transcription factors, and these domains produce progenitors for specific subsets of OB interneurons (Figure 1; see Fiorelli et al., 2015) for a recent review].

Calretinin-expressing interneuron progenitors are generated from the dorsal and medial walls of the subventricular zone that are marked by Sp8 expression (Merkle et al., 2007, 2014; Young et al., 2007; Fernández et al., 2011). The conditional loss of Sp8 severely reduces the number of interneurons that contain Calretinin (Waclaw et al., 2006). In mature Calretinin interneurons, Sp8 expression is maintained and is partially co-expressed Meis2 (Allen et al., 2007).

By contrast, dopaminergic progenitors are generated from a dorso-lateral region of the subventricular zone marked by the expression of Pax6 (Merkle et al., 2007; Young et al., 2007; Brill et al., 2008; Fernández et al., 2011). Pax6 is necessary for the dopaminergic phenotype (Dellovade et al., 1998; Kohwi et al., 2005; Brill et al., 2008; Haba et al., 2009), and works in combination with the Dlx2 and Meis2 transcription factors (Brill et al., 2008; de Chevigny et al., 2012b; Agoston et al., 2014). Pax6 remains expressed in terminally differentiated dopaminergic neurons and is required for their survival (Ninkovic et al., 2010).

Progenitors for interneurons containing Calbindin arise from ventral regions of the subventricular zone (Merkle et al., 2007, 2014; Young et al., 2007; Brill et al., 2008; Fernández et al., 2011). Mature Calbindin-containing interneurons in mice co-express Meis2, but Meis2 is broadly expressed in several OB interneuron subsets (Allen et al., 2007) and transcription factors that are preferentially expressed with Calbindin remain to be identified.

In contrast to rodents, few studies have examined transcription factor codes underlying the differentiation of human OB interneuron phenotypes. Recent studies indicate that an important fundamental difference between the adult rodent and adult human OB is the turnover and regeneration of interneurons. In rodents, there is continuous turnover and regeneration of OB interneurons by SVZ-derived progenitors throughout the lifetime of the animal (Cave and Baker, 2015). By contrast, human OB interneurons appear to be generated during late gestation and early post-natal periods, after which the production of OB interneuron progenitors in the SVZ ceases (Sanai et al., 2004, 2011; Quiñones-Hinojosa et al., 2006; Wang et al., 2011; Bergmann et al., 2012). This difference raises the question of whether there is conservation in the molecular mechanisms that organize the OB in rodents and humans, which includes the question of whether the transcription codes that specify interneuron phenotypes are conserved between the species. Given that rodents are the most common experimental model system for humans, it is essential to establish that the transcription codes generated in rodents are conserved in humans. In this study, we examined a select group of transcription factors to establish whether their co-expression with Calretinin, Calbindin, or Tyrosine hydroxylase has been conserved between mice and humans.

## Materials and methods

### Mice

All mice in this study were adults on a C57BL6 background that were between 6 and 10 months of age. Both male and female mice were used, and all mice were housed in humidity-controlled cages at 22°C under a 12:12 h light/dark cycle and provided with food and water *ad libitum*. All procedures were carried out under protocols approved by the Weill Cornell Medical College Institutional Animal Care and Use Committee and conformed to NIH guidelines.

### Human olfactory bulb tissue

Human OB tissue was collected post-mortem by the Harvard Brain Tissue Resource Center. All patient and informed consent data was collected and maintained by the Harvard Brain Tissue Resource Center. All subjects in this study were males that presented no sign of neurodegenerative disease either before death (such as a clinical diagnosis or a documented symptoms consistent with tremor or impairment of cognition) or during autopsy (such as abnormal atrophy within the brain). All available information regarding donor subjects is shown in Table 1. Human tissue work at the Burke Medical Research Institute was approved by the Burke Rehabilitation Hospital Committee for Human Rights in Research.

**Table 1:** **Donor subject information for the tissue examined in this study**.

**Subject ID**	**Sex**	**Age**	**Post Mortem Interval (hours)**
8108	M	70	31.6
8314	M	90	21.9
8358	M	76	24.2

### Tissue preparation and immunofluorescence

All mouse tissue was from animals transcardially perfused with 4% paraformaldehyde, and was post-fixed for 2 h in 4% paraformaldehyde before being cyroprotected overnight in 30% sucrose. All human tissues were formalin-fixed for the duration between harvest and arrival in laboratory (16–24 h), after which the tissue was cyroprotected overnight in 30% sucrose. Both mouse and human tissue was cut as 25 μm thick sections on a cryostat. For immunofluorescent staining, sections were incubated overnight with primary antibodies diluted in phosphate buffered saline. Primary antibody manufacturers and dilutions are provided in Table 2. Sections were then washed and incubated for 1 h with AlexaFluor (488, 594, or 647) secondary antibodies (Life Technologies) that were diluted at 1:400 in phosphate buffered saline. After removing the secondary antibodies, mouse sections were washed, dried and cover-slipped using Prolong Gold with DAPI (Life Technologies). Human sections were immersed in 0.02% Sudan Black in 70% ethanol for 20 min, then rinsed with 70% ethanol before being cover-slipped using Prolong Gold with DAPI (Life Technologies).

**Table 2:** **Primary antibodies used in this study**.

**Antigen**	**Source**	**Host species**	**Dilution**
Tyrosine Hydroxylase (Th)	T. Joh (Weill Cornell Medical College) (Joh et al., 1973)	Rabbit	1:2500
Tyrosine Hydroxylase (Th)	Immunostar (22941)	Mouse	1:1000
Calretinin	EMD Millipore (AB5054)	Rabbit	1:1000
Calretinin	EMD Millipore (AB1550)	Goat	1:2500
Calbindin	EMD Millipore (AB1778)	Rabbit	1:1000
Calbindin	Sigma-Aldrich (CB-955)	Mouse	1:1000
Foxp2	Abcam (ab16046)	Rabbit	1:1000
Meis2	A. Buchwald (Thomas Jefferson Univ.) (Swift et al., 1998)	Rabbit	1:500
Pax6	Sigma-Aldrich (HPA030775)	Rabbit	1:2000
Sp8	Santa Cruz (sc-104661)	Goat	1:2000

### Imaging and data analysis

All sections were imaged on Nikon 80i Eclipse and Zeiss 200M Axiovert fluorescent microscopes. A minimum of three individual mice and two human subjects were analyzed for each antigen pairing. Antigen co-expression is reported as a percentage of the total number of cells containing either Th, Calretinin, or Calbindin that co-expressed the transcription factor of interest. The total number of cells counted for each interneuron phenotype in mice and humans is listed in Table 3.

**Table 3:** **Total number of cells counted of each interneuron phenotype for co-expression with the corresponding transcription factor**.

**Interneuron phenotype**	**Foxp2**	**Meis2**	**Pax6**	**Sp8**
Calbindin (mouse)	1216	665	660	1515
Calretinin (mouse)	1345	1208	3515	1445
Th (mouse)	867	741	749	1096
Calbindin (human)	14	22	17	136
Calretinin (human)	2845	1853	1596	1499
Th (human)	522	293	968	489

## Results

### Distribution of calbindin, TH, and calretinin in the mouse and human OB

**Figure 2:**
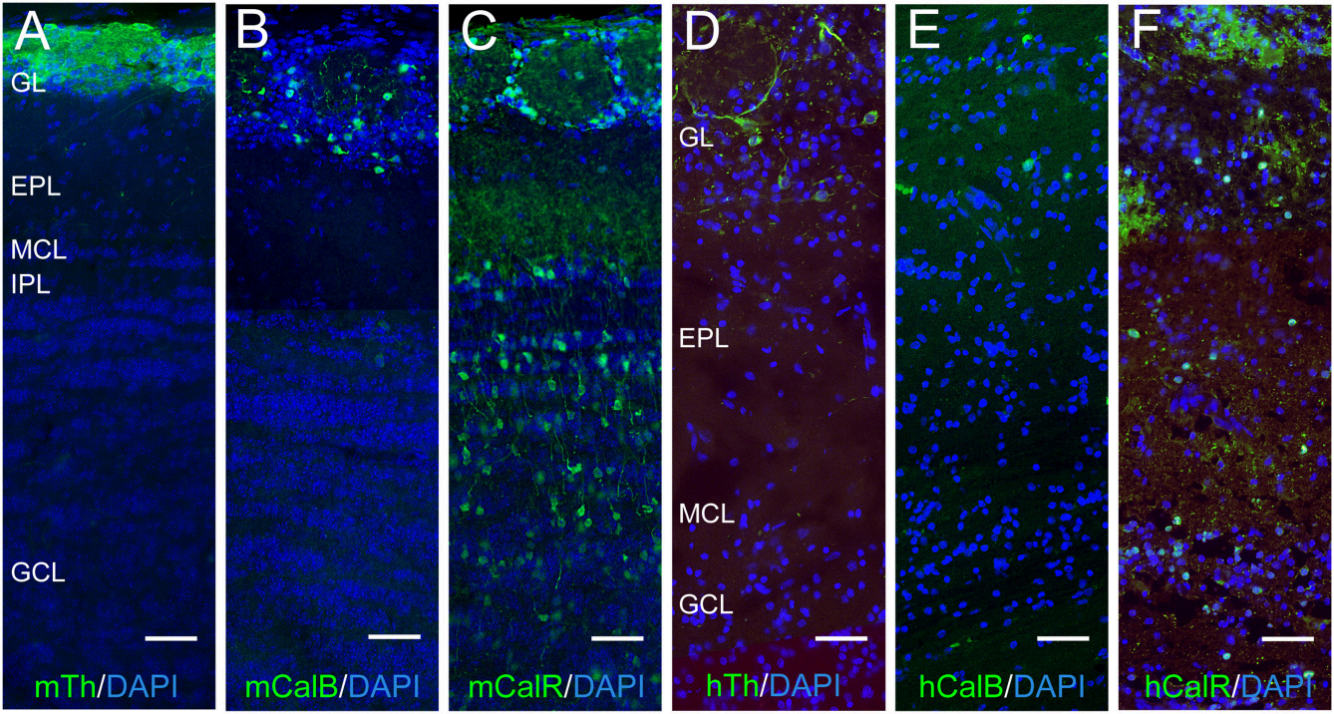
**Laminar organization of Th, Calbindin, and Calretinin expression in the mouse and human OB**. **(A–C)** and **(D–F)**, Th, Calbindin and Calretinin expression (green) in the mouse (m) and human (h) OB, respectively. Nuclei are labeled with DAPI (blue). Abbreviations: GL, glomerular layer; EPL, external plexiform layer; MCL, mitral cell layer; IPL, internal plexiform layer; GCL, granule cell layer. Bar = 100 μm.

In the mouse OB, Calbindin, and Th expression was restricted to the glomerular layer (Figures 2A,B). By contrast, Calretinin was expressed in both the glomerular and granule cell layers (Figure 2C). These expression patterns were consistent with previous descriptions (see Parrish-Aungst et al., 2007 and references therein). Th expression in the human OB was similar to the mouse and confined to the glomerular layer (Figure 2D), as previously reported (Smith et al., 1991). Calbindin-expressing interneurons in the human OB were scarce, and were largely found in the glomerular and external plexiform layers (Figure 2E). Larger Calbindin-expressing cells were also present in the anterior olfactory nucleus and the lateral olfactory tract (not shown), but these cells were beyond the scope of the current study and not examined further. This expression pattern for Calbindin in the human OB was consistent with a previous description (Ohm et al., 1991). Calretinin-expressing cells in the human OB were present in the glomerular, external plexiform, and granule cells layers (Figure 2F). Strong Calretinin expression was also observed in the olfactory sensory axon terminals in the glomerular layer (Figure 2F). The laminar distribution of Calretinin in the human OB has not been previously reported, but the expression pattern observed in this study is similar to a previous report with the *Macaque* monkey (Alonso et al., 2001).

### Distribution of Meis2, Pax6, Sp8, and Foxp2 in the mouse and human OB

**Figure 3:**
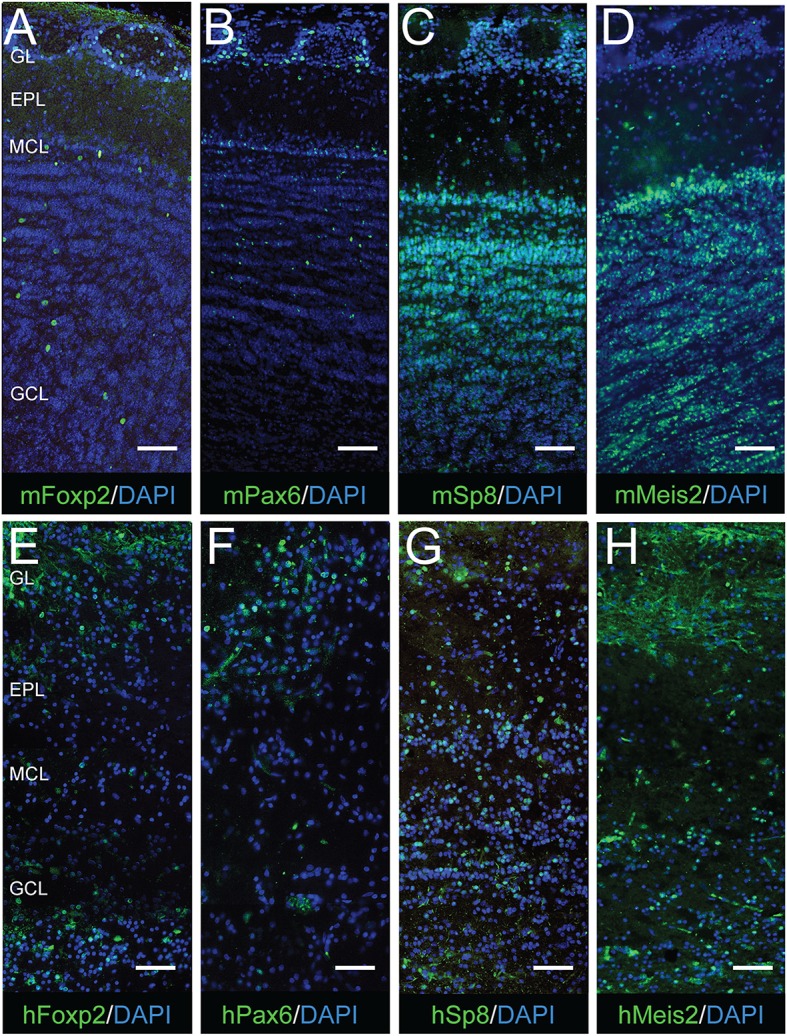
**Laminar organization of Foxp2, Pax6, Sp8, and Meis2 expression in the mouse and human OB**. **(A–D)** and **(E–H)**, Foxp2, Pax6, Sp8, and Meis2 expression (green) in the mouse (m) and human (h) OB, respectively. Nuclei are labeled with DAPI (blue). Abbreviations: GL, glomerular layer; EPL, external plexiform layer; MCL, mitral cell layer; IPL, internal plexiform layer; GCL, granule cell layer. Bar = 125 μm.

Based on studies in mice: Pax6 was examined because of its preferential co-expression with Th (Allen et al., 2007; Baltanás et al., 2009); Sp8 was examined because of its strong co-expression with Calretinin (Allen et al., 2007; Kosaka and Kosaka, 2012); and Meis2 was examined because of its broad expression and co-expression with Calbindin, Th, and Calretinin (Allen et al., 2007). In both the mouse and human OB, Pax6 expression was concentrated in the glomerular layer with scattered cells in the external plexiform and granule cell layers also showing immunoreactivity (Figures 3B,F). This pattern was consistent with previous studies in mice (Dellovade et al., 1998). In contrast to Pax6, Sp8-expressing cells were numerous in all of the lamina in both mice and humans (Figures 3C,G). In the mouse granule cell layer, however, Sp8 expression was noticeably more intense in the superficial region. These observations were consistent with previous studies in both mice and humans (Waclaw et al., 2006; Wang et al., 2014). Meis2 also showed broad expression in all OB lamina of humans and mice (Figures 3D,H). Although Meis2 was expressed in most glomerular layer cells in mice, its intensity was lower than the labeled cells in the granule cell layer. This broad expression pattern matched previous reports in mice (Allen et al., 2007).

**Figure 4:**
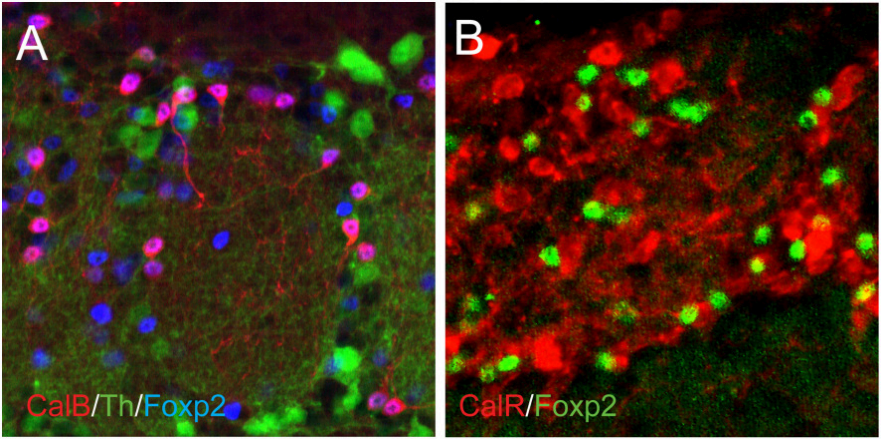
**Foxp2 co-expression with Th, Calbindin, and Calretinin in the mouse OB**. **(A)**, Calbindin (red) showed strong co-expression with Foxp2 (blue), but Th (green) showed little or no overlapping expression with Foxp2 (blue). **(B)**, Calretinin (red) also showed no overlapping expression with Foxp2 (green).

The transcription factor Foxp2 was examined based on previous reports of its expression in the lateral wall of the subventricular zone and olfactory bulb (Ferland et al., 2003; Campbell et al., 2009; Azim et al., 2015). This study found that nearly all cells expressing Foxp2 in the mouse OB were in the glomerular layer, and only a few cells in either the external plexiform layer or granule cell layer also showed Foxp2 expression (Figure 3A). This pattern was similar to a previous examination of adult mice (Campbell et al., 2009). In the human OB, Foxp2-expressing cells were also largely in the glomerular layer, but the proportion of Foxp2-expressing cells in the external plexiform and granule cell layers was considerably greater (Figure 3E). Whether Foxp2 is co-expressed with Th, Calretinin, or Calbindin has not been previously addressed. In this study, strong Foxp2 co-expression with Calbindin was observed in the adult mouse OB (Figure 4A). This co-expression was selective for Calbindin since neither Th nor Calretinin showed substantial overlap with Foxp2 (Figures 4A,B). Foxp2 expression was not exclusive to Calbindin-containing cells in the OB, however, as there were several Foxp2-labeled cells that did not co-express Calbindin (Figure 4A). Together, these findings revealed that Foxp2 was a novel marker of the Calbindin interneuron phenotype in the mouse OB.

### Transcription factor expression in interneuron phenotype subsets

**Figure 5:**
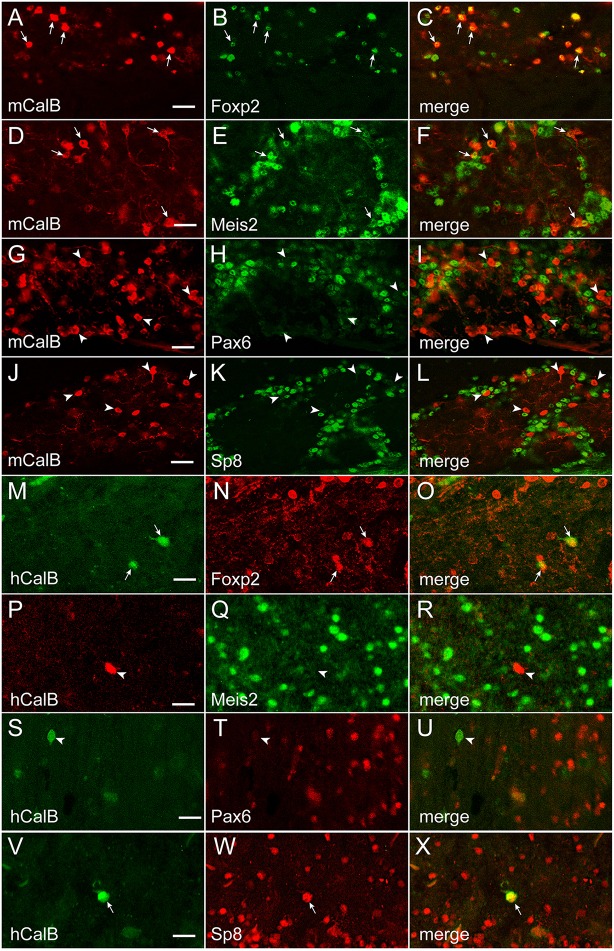
**Transcription factor co-expression with Calbindin in the OB glomerular layer**. **(A–L)** and **(M–X)**, Calbindin co-expression with Foxp2, Meis2, Pax6, or Sp8 in the in the mouse (m) and human (h) OB, respectively. Arrows mark cells that co-expressed Calbindin and the transcription factor, whereas arrowheads mark Calbindin-expressing cells that lacked the transcription factor. Bar = 50 μm.

**Figure 6:**
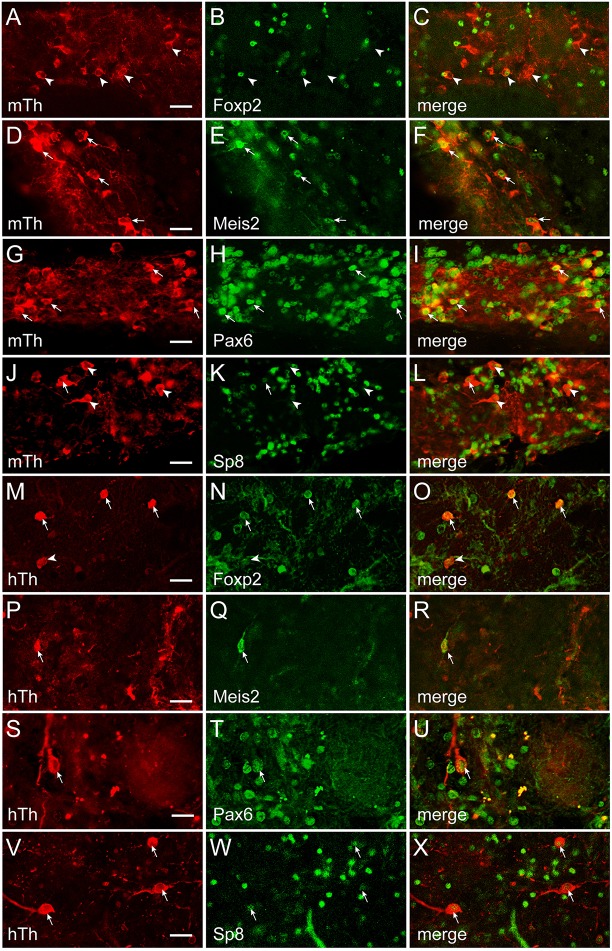
**Transcription factor co-expression with Th in the OB glomerular layer**. **(A–L)** and **(M–X)**, Th co-expression with Foxp2, Meis2, Pax6, or Sp8 in the in the mouse (m) and human (h) OB, respectively. Arrows mark cells that co-expressed Th and the transcription factor, whereas arrowheads mark Th-expressing cells that lacked the transcription factor. Bar = 50 μm.

**Figure 7:**
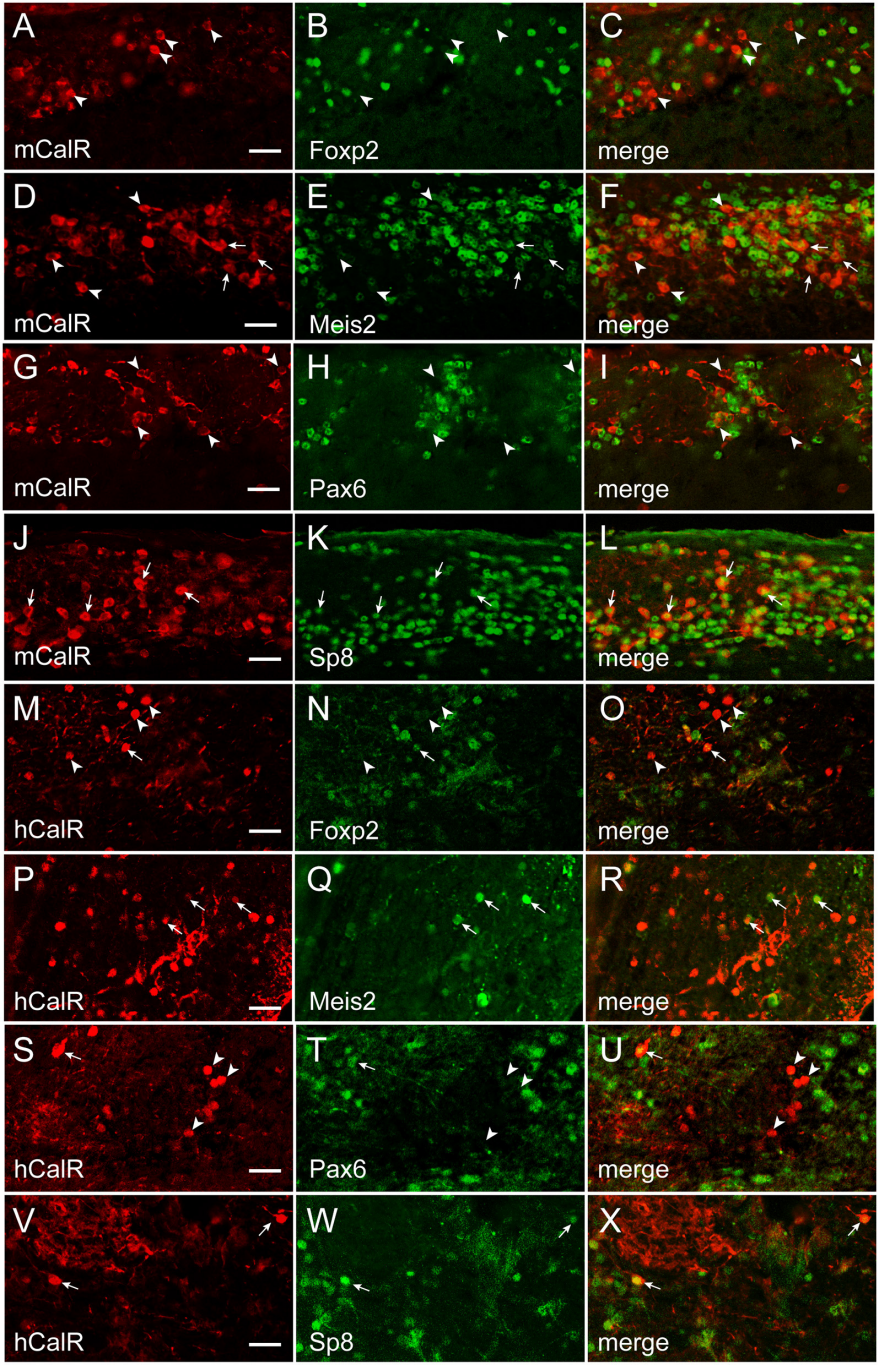
**Transcription factor co-expression with Calretinin in the OB glomerular layer**. **(A–L)** and **(M–X)**, Calretinin co-expression with Foxp2, Meis2, Pax6, or Sp8 in the in the mouse (m) and human (h) OB, respectively. Arrows mark cells that co-expressed Calretinin and the transcription factor, whereas arrowheads mark Calretinin-expressing cells that lacked the transcription factor. Bar = 50 μm.

To establish whether transcription factor codes were conserved between humans and mice, the co-expression pattern for each transcription factor and interneuron phenotype marker was examined by immunofluorescence (Figures 5–7). The extent of co-expression was measured as the percentage of each interneuron phenotype that co-expressed the transcription factor of interest (Table 4). For Calbindin-expressing interneurons, Foxp2 was strongly co-expressed in both mice and humans (Figure 5). In contrast, Pax6 showed little association with Calbindin. Meis2 and Sp8, however, were differentially co-expressed with Calbindin. Meis2 was strongly co-expressed in mice, but had only weak co-expression in humans. In contrast, Sp8 co-expression was high in humans, but largely absent in mice. For dopaminergic interneurons, strong Meis2, and Pax6 co-expression with Th was conserved between mice and humans (Figure 6). Human dopaminergic interneurons also showed high levels of co-expression with Foxp2 and Sp8, but these transcription factors showed weak co-expression in the mouse OB. Unlike either Th or Calbindin, transcription factor co-expression profiles with Calretinin did not show any species-specific differences (Figure 7). Sp8 was highly co-expressed with Calretinin, and the majority of Calretinin-containing cells also expressed Meis2. In both species, co-expression with either Foxp2 or Pax6 was weak. Specific percentages of interneuron subtypes co-expressing one or more of the different transcription factors described above are reported in Table 4.

**Table 4:** **The percentage of each interneuron phenotype that co-expressed the transcription factor of interest**.

	**Percent of Calbindin-expressing intereuron co-expressing the corresponding transcription factor**		**Percent of Calretinin-expressing intereuron co-expressing the corresponding transcription factor**		**Percent of Th-expressing intereuron co-expressing the corresponding transcription factor**	
**Transcription factor**	**Mouse**	**Human**	**Mouse**	**Human**	**Mouse**	**Human**
Foxp2	96	71	1	21	9	75
Meis2	92	11	62	76	90	86
Pax6	3	18	2	16	98	80
Sp8	6	84	95	86	18	91

## Discussion

**Figure 8:**
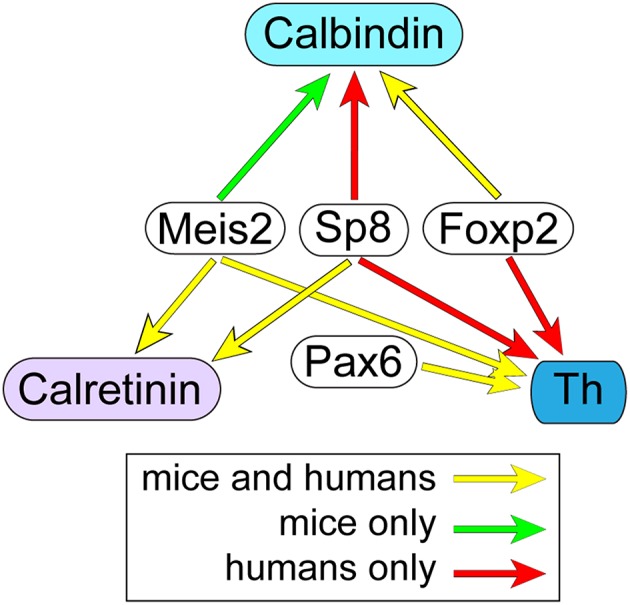
**Conservation of OB interneuron transcription codes between humans and mice**. Yellow arrows indicate transcription factors with co-expression in at least 60% of interneurons containing either Th, Calbindin, or Calretinin that was conserved between humans and mice. By contrast, red and green arrows indicate transcription factors that were co-expressed in at least 60% of interneurons containing either Th, Calbindin, and Calretinin in only humans and mice, respectively.

The co-expression analyses in this study identified OB interneuron transcription codes that are conserved between the mice and humans (summarized in Figure 8). For the OB dopaminergic phenotype, strong co-expression with Pax6 was conserved between mice and humans. Studies in rodents have shown that Pax6 is necessary for this specifying phenotype (Dellovade et al., 1998; Kohwi et al., 2005; Brill et al., 2008; Haba et al., 2009), and regulation of Pax6 expression by miR-7a is important for fine-tuning the ability of Pax6 to direct phenotype specification (de Chevigny et al., 2012a). Rodents studies have also shown that Pax6 over-expression in OB progenitors is sufficient to specify the OB dopaminergic phenotype (Hack et al., 2005). The conservation of the strong co-expression with Pax6 and Th suggests that Pax6 also has a central role in specifying the human OB dopaminergic phenotype. The present study also showed strong co-expression between Meis2 and Th in mice and humans, which indicates that a combinatorial code of Meis2 and Pax6 for specifying OB dopamine neurons is conserved between species. Recent studies in mice showed these two proteins physically interact and activate *Th* transcription by binding to an upstream site (Agoston et al., 2014). The transcription factor Nurr1 (Nr4a2) directly regulates transcription of a dopaminergic gene battery, which includes *Th* (Jacobs et al., 2009a,b), and whether Meis2 and Pax6 also target other genes in the Nurr1-regulated dopaminergic gene battery in the OB remains to be established. The analysis of Sp8 co-expression with Th suggests that there are subtypes of OB dopaminergic neurons, and the proportion of these subtypes is species-dependent. The current study found that only about 20% of Th-containing cells in the mouse OB co-expressed Sp8, which is similar to a previous report (Allen et al., 2007). This level of co-expression is consistent with an approximate 20% reduction in the number of OB Th-expressing cells in Sp8 conditional knock-out (Waclaw et al., 2006). Kosaka and Kosaka, however, reported most mouse OB dopaminergic neurons express Sp8 at low levels (Kosaka and Kosaka, 2012). The conditional Sp8 knock out findings suggest that for most mouse OB dopaminergic neurons, low Sp8 expression levels are not required for their differentiation. In humans, however, this study found that the vast majority of OB dopaminergic neurons co-expressed Sp8, which is consistent with reports from rhesus monkeys (Wang et al., 2014). This suggests that the combinatorial code of Meis2 and Pax6 is required for the general dopaminergic phenotype, but there may be at least two subtypes of OB dopaminergic neurons that are specified by differential expression levels of Sp8. Our findings indicate that the specification of most human OB dopaminergic neurons requires high Sp8 expression, but most mouse OB dopaminergic neurons have low Sp8 expression levels and only a minor population is specified with high Sp8 levels. Previous studies in both rodents and humans have suggested that OB dopaminergic neurons are not homogenous and there are multiple subtypes based on morphology (Halasz et al., 1981; Hoogland and Huisman, 1999; Kosaka and Kosaka, 2009; Kiyokage et al., 2010). A challenge for future studies is to establish whether the morphological subtypes correlate with the differential expression levels of either Sp8 or other transcription factors that also specify the OB dopaminergic neurons, including Etv1, Zic1/3, Sall3, COUP-TFI, and Dlx2 (Stenman et al., 2003; Inoue et al., 2007; Brill et al., 2008; Harrison et al., 2008; Bovetti et al., 2013).

For the Calretinin phenotype, the strong conservation of Sp8 co-expression indicates that this transcription factor is an important determinant of this interneuron phenotype. Sp8 expression by itself, however, is not sufficient to drive Calretinin gene expression since Sp8 was also strongly co-expressed with Th in humans, and Th and Calretinin expression is mutually exclusive (Figure S1). Specification of the Calretinin phenotype by Sp8 may require coordination with COUP-TFI/II transcription factors, since OB Calretinin interneuron production is disrupted in conditional COUP-TFI/II mutant mice (Zhou et al., 2015). Alternatively, or in addition, Sp8 may interact with the Wnt signaling pathway to selectively target the Calretinin promoter. The Wnt signaling pathway directly activates Calretinin expression in thalamic neurons (Wisniewska et al., 2012), and recent studies have shown that Sp8 is a gene-specific transcriptional coactivator in the Wnt/β-catenin pathway (Kennedy et al., 2016). In addition to Sp8, co-expression of Meis2 in about 65–75% of Calretinin-expressing cells was also conserved between mice and humans. Like the dopaminergic interneurons, this suggests that there are at least two subtypes of Calretinin-containing interneurons in the OB based on the differential co-expression of Meis2. The consequences of the differential co-expression of Meis2 in these cells is unclear, but may indicate that the two subtypes are generated in different SVZ progenitor domains. Alternatively, the differential co-expression may result in the expression of alternative gene expression profiles that endow the subtype with different functional properties.

This study showed that Foxp2 is a novel marker for the Calbindin OB interneuron phenotype, and the conservation of this co-expression suggests that its role in specifying this phenotype is shared between mice and humans. Other factors that work in combination with Foxp2 to specify the OB Calbindin phenotype are not known, but Tshz1 is one possible factor since its conditional loss in neural progenitors significantly reduces the number of Calbindin-expressing neurons in the OB (Ragancokova et al., 2014). Alternatively, or in addition, Foxp2 may interact with factors related to the Sonic hedgehog (Shh) signaling pathway. Shh is present in the ventral region of the SVZ, and the conditional loss of Shh signaling strongly reduces the generation of Calbindin-expressing interneurons in the adult OB (Ihrie et al., 2011). The strength of Shh in the ventral SVZ may be an important determinant of the Calbindin phenotype, as it is in the specification of cortical interneuron progenitor fates (Xu et al., 2010). Foxp2 may also interact with downstream targets of the Shh pathway, such as Nkx2-1. Shh signaling induces Nkx2-1 expression (Ruiz i Altaba, 1998; Gulacsi and Anderson, 2006), and the ventral region of the SVZ is of an Nkx2-1 lineage (Xu et al., 2008). Foxp2 has been reported to regulate specification of striatal interneurons by interacting with the Shh/Nkx2-1 pathway (Chiu et al., 2014), and may have a similar regulatory role in both the post-natal and adult SVZ.

Together, the findings in this study provide important insight into the conservation of transcription factor codes for OB interneuron phenotypes between humans and mice. Further studies examining the co-expression of additional transcription factors with Th, Calretinin, and Calbindin will help to refine these codes and detail how these phenotypes are specified. In addition, studies examining transcription factor co-expression patterns at different ages in mice could address whether the relative proportion of subsets within a specific phenotype (such as high vs. low Sp8 expression in dopaminergic interneurons) changes as a function of age. This type of age-dependent analysis could address whether the transcription factor codes in the human OB resemble the young post-natal mouse or if the variation between mice and humans observed in this study reflect inherent differences between the species. These OB interneuron transcription factor codes provide an important complement to codes that have been established for neuronal subtypes derived from other regions within the mammalian central nervous system, such as the cortex and spinal cord (Jessell, 2000; Shirasaki and Pfaff, 2002; Gelman and Marín, 2010; Arber, 2012; Woodworth et al., 2012; Nord et al., 2015).

## Author contributions

NF performed experiments and analysis; JC designed the study, performed experiments and data analysis, and wrote the manuscript.

## Funding

Funding was provided by the National Institutes of Health (DC008955 and MH068855) and the Burke Medical Research Institute.

### Conflict of interest statement

The authors declare that the research was conducted in the absence of any commercial or financial relationships that could be construed as a potential conflict of interest.
