# Supplementary material for: Partial Conservation between Mice and Humans in Olfactory Bulb Interneuron Transcription Factor Codes
Source: Front Neurosci. 2016 Jul 20;10:337. doi: 10.3389/fnins.2016.00337 (PMC4951497; doi:10.3389/fnins.2016.00337)
Supplement: Supplementary file 1 [file Image1.pdf]

Supplementary Material

Partial Conservation Between Mice and Humans in Olfactory Bulb of Interneuron Transcription Factor Codes

Nana Fujiwara, John W. Cave*

*** Correspondence:** John W. Cave: joc2042@med.cornell.edu

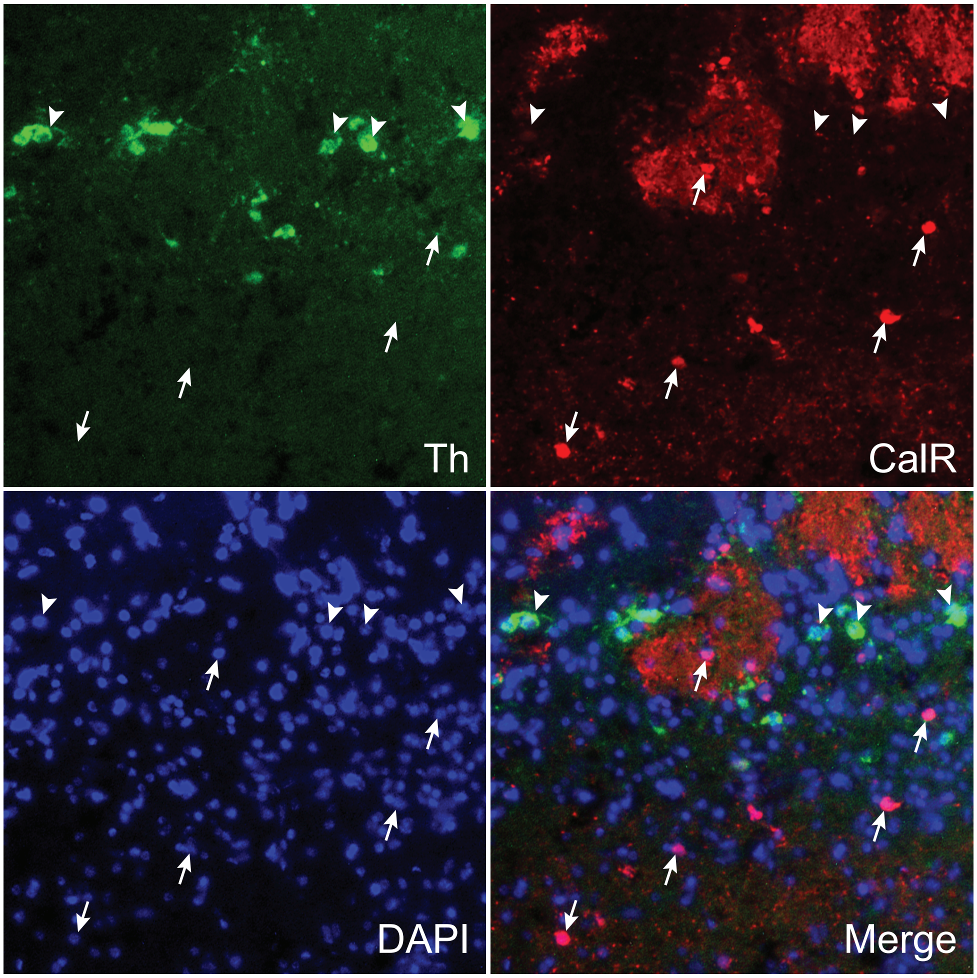

1 Supplementary Figure

Supplementary Figure S1. Th and Calretinin expression in the adult human olfactory bulb is mutually exclusive. Arrowheads indicate the position of Th-expressing cells, and arrows mark the position of cells containing Calretinin.
